# The implementation and impact of a pilot hydrocele surgery camp for LF-endemic communities in Ethiopia

**DOI:** 10.1371/journal.pntd.0009403

**Published:** 2021-10-25

**Authors:** Andualem Deneke Beyene, Fikreab Kebede, Belete Mengistu Mammo, Biruck Kebede Negash, Addisalem Mihret, Solomon Abetew, Asfaw Kejella Oucha, Shigute Alene, Sharone Backers, Sunny Mante, Zeina Sifri, Molly Brady, Scott McPherson

**Affiliations:** 1 Department of Surgery, School of Medicine, Addis Ababa University, Addis Ababa, Ethiopia; 2 RTI International, Addis Ababa, Ethiopia; 3 Federal Ministry of Health, Addis Ababa, Ethiopia; 4 Regional Health Bureau, Beneshangul-Gumuz, Ethiopia; 5 RTI International, Beneshangul-Gumuz, Ethiopia; 6 African Filariasis Morbidity Project, Accra, Ghana; 7 Helen Keller International, Washington DC, United States of America; 8 RTI International, Washington DC, United States of America; Instituto de Ciências Biológicas, Universidade Federal de Minas Gerais, BRAZIL

## Abstract

**Background:**

Ethiopia aims to eliminate lymphatic filariasis by 2020, through a dual approach of mass drug administration to interrupt transmission and morbidity control which includes making hydrocele surgery available in all endemic areas. Locating patients requiring surgery, providing high quality surgeries, and following up patients are all formidable challenges for many resource-challenged or difficult-to-reach communities. To date, hydrocele surgery in Ethiopia has only occurred when a patient has the knowledge, time and resources to travel to regional hospitals. Ethiopia tested the novel approach of using a surgical camp, defined as mobilizing, transporting, providing surgery at a static site, and following up of a large cohort of hydrocele patients within a hospital’s catchment area, to address delays in seeking and receiving care.

**Methodology and results:**

Health extension workers mobilized 252 patients with scrotal swelling from a list of 385 suspected hydrocele cases from seven endemic districts in the region of Beneshangul-Gumuz. Clinical health workers and surgeons confirmed 119 as eligible for surgery. Of 70 additional patients who self-referred, 56 were eligible for surgery. Over a two-week period at a regional hospital, 175 hydrocele excision surgeries were conducted. After discharge three days after surgery, trained clinical health workers followed up with the patients on Day 5, Day 8, Day 14 and 1st-month benchmarks with a randomized follow-up of a selection of patients conducted at 9–12 months. There were no post-operative complications upon discharge at Day 3 and 22, while minor complications occurred (12.6%) between Day 3 and one month. The 9–12 month follow-up found patients self-reported an improvement in quality of life, health and economic status.

**Conclusion:**

A hydrocele surgery camp was effective at providing a large number of quality surgeries in a short time. Using peripheral health workers to mobilize and follow up patients helped address delays in seeking and receiving quality care. Mainstreaming patient mobilization and follow-up into a community health system could be effective in other countries. The camp’s results also influenced two regions in Ethiopia to change their policies in order to offer free hydrocele surgery (including patient transport, consultation, surgery, diagnostic tests and necessary medications).

## Author summary

Hydrocele is a type of scrotal swelling caused by lymphatic filariasis and requires surgery to treat, which can be challenging to address in many endemic countries. Locating and referring patients, providing high quality surgical intervention, and creating a patient follow-up mechanism are all formidable aspects in communities that are often resource challenged and difficult to reach. We explored if the use of a surgical camp setting would be an appropriate intervention to adequately treat a large number of hydrocele patients over a short period, decreasing delays in seeking, reaching and receiving quality surgery for hydrocele. We analyzed each step required to hold the camp including some novel innovations surrounding patient mobilization, referral, and post-surgical quality assurance. The camp was effective at providing a large number of quality surgeries in a short time, with patients self-reporting improvements in quality of life, economic and health status after surgery. The findings from the study may prove useful for countries seeking to implement similar interventions.

## Introduction

Lymphatic filariasis (LF) is a vector-borne parasitic disease slated by the World Health Organization (WHO) for elimination as a public health problem by 2020 [1]. WHO has two elimination strategies: reducing transmission through annual mass drug administration; and reducing suffering through ensuring the availability of morbidity management and disability prevention services to all patients [1]. Infection with the parasite causes damage to the lymphatic system and can result in chronic clinical conditions including hydrocele, or scrotal swelling, and lymphedema, or swelling of the arms, legs or breasts [2]. In 2013 it was estimated that, worldwide, more than 19.4 million males suffer from hydrocele, and more than 16.7 million people suffer from lymphedema [3]. Hydrocele is caused by an imbalance of fluids secreted and absorbed by the tunica vaginalis due to damage to the lymphatic system from the parasites [4]. It causes disfigurement, loss of libido, erectile dysfunction, loss of the ability to walk and work, and the erosion of a patient’s surrounding social structure [5–7].

Hydrocele surgery is recognized by WHO as an essential surgery [8], either using the eversion technique, in which the edges of the tunica vaginalis are everted behind the testis and sutured together, or the excision technique, in which the parietal layer of the tunica vaginalis is excised, leaving 1–2 centimeters width at the edge of the testis. The WHO LF elimination strategy includes providing surgery for hydrocele patients through the health system in a long-term, affordable and sustainable manner, with goals of including surgery in universal health coverage essential packages and providing surgery without out-of-pocket expenses for patients by 2030 [9,10]. Ensuring high quality surgical interventions are available to patients has been difficult in resource-challenged areas given cost, access, and follow-up issues. Hydrocele surgical camps are concentrated, time-bound initiatives that bring surgical services to a specific location to target large numbers of patients with suspected hydrocele, and that have been conducted successfully in the past in Africa [11]. However, little has been published on surgical best practices and outcomes of hydrocele surgery in LF-endemic areas [2,4,5,8,11–13]. This paper reports on the medical and self-reported socio-economic outcomes of a hydrocele surgery training and camp conducted in Ethiopia in February 2017.

Ethiopia has a paucity of surgeons and surgery capacity with the lowest measured rate of surgery in the world [14]. Although over 5 million surgical interventions are estimated to be needed each year, fewer than 200,000 surgeries (4%) actually occur [15]. There are currently an estimated three general surgeons per one million population in Ethiopia, with 48% of these surgeons practicing in Addis Ababa [16], well below the global target of 20 surgical, anesthesia, and obstetric providers per 100,000 people [17]. Relatedly, while 97% of tertiary hospitals in Ethiopia report providing hydrocele surgeries [18] a hydrocele surgery readiness analysis conducted in three regions showed that at most five hydrocele surgeries were conducted per month per visited tertiary hospital.

The Federal Ministry of Health (FMOH) aims to increase access to surgical services including hydrocele surgery at all hospitals by 2020 as part of its Saving Lives Through Safe Surgery (SaLTS) initiative [19]. To do so, it must overcome what the Lancet Surgical Commission coins the ‘three delays’ in accessing appropriate surgical care: i) delay in seeking care because of isolation and low awareness of available services, ii) delay in reaching care because of distance from and cost to reach health facilities, and iii) delay in receiving care due to lack of trained surgical staff [17].

Delays in seeking hydrocele surgery can occur because insufficient patient outreach is conducted. Delays in reaching care occur because hydrocele surgery is not included in insurance schemes, making it cost prohibitive. In addition, hydrocele surgery is normally performed as an outpatient procedure using local anesthesia with same-day discharge in Ethiopia, resulting in patients who require several days’ journey to the hospital not reporting back for the nine post-operative appointments recommended to monitor complications and recurrence [20]. Delays in receiving care occur in part due to the scarcity of surgeons, which Ethiopia has begun to address through creating an additional cadre of integrated emergency surgical officers (IESOs). As of July 2016, 287 IESOs, who receive a three-year training on emergency and essential surgery, gynecological and obstetrics procedures including eversion and excision techniques for hydrocele surgery, had been trained to complement the 277 practicing general surgeons [21]. The pilot hydrocele surgery camp intervention discussed in this article aimed to further address all three of the delays specified by the Lancet Commission’s report.

## Methods

### Ethics statement

This pilot hydrocele surgery camp intervention aimed to demonstrate whether a surgery camp can provide a high number of hydrocele surgeries with good outcomes, as measured by low post-operative complications and improved Quality of Life of participants. A ‘hydrocele surgery camp’ was defined as mobilizing, providing surgery for, and following up a large cohort of hydrocele patients within a hospital’s catchment area within two weeks.

The FMOH and Beneshangul-Gumuz Regional Health Bureau approved implementation of the training and the camp. The Beneshangul-Gumuz Regional Health Bureau Ethical Committee reviewed and approved the patient follow-up protocol. Individual patients signed written consent forms before surgery after nurses counselled them on the procedure. All patient medical records were maintained by Assosa hospital according to the patient privacy laws of Ethiopia, and the study anonymized individual patients during subsequent data analysis and write-up.

### Study site

At the time of this 2017 pilot, Ethiopia had completed hydrocele patient estimate exercises in 52 of the 70 (74%) LF-endemic woredas (i.e., districts or third-level administrative divisions of Ethiopia) in the country resulting in the detection of 1,889 suspected hydrocele cases. These estimates were completed predominantly through house-to-house visits that were either stand-alone or conducted during pre-LF MDA registration. Beneshangul-Gumuz region, which had 12 LF-endemic woredas with patient estimates, was chosen for this study because it contained the highest known number of suspected hydrocele cases. A five-day hydrocele surgery training of 20 general surgeons and integrated emergency surgical officers and a subsequent five-day camp were conducted at the tertiary hospital in the regional capital, Assosa, whose catchment area included seven of the 12 LF-endemic woredas closest to Assosa town, i.e. Asossa, Homosha, MaoKomo, Menge, Oda Bildigilu, Sedal (Sirba Abay), and Sherkole. An estimated 385 suspected hydrocele patients were targeted for pre-screening before the camp, addressing 81% (385/473) of the total cases of scrotal swelling identified in the 12 LF-endemic woredas in the region.

In October 2016, in preparation for the camp, two study organizers, with hospital administrators and local health officials, implemented a readiness assessment at Assosa hospital to ensure the necessary infrastructure and human resources were available. The assessment identified five available operating tables in four theaters, with capacity to perform a total of up to 50 hydrocele surgeries per day based on an estimated average time per surgery per theater, as well as sufficient quantities of surgical sets, drugs, consumables, and basic laboratory tests (urinalysis, haemoglobin, blood group, HIV status). The hospital had adequate sterilization and cleaning capabilities, and a generator available for backup. Local human resources included a dedicated scrub nurse for each of the operating tables, anaesthetists, circulation nurses for immediate post-operative care, and a general administrator to coordinate these activities around the regular daily duties of the hospital. Finding space and bedding for the patients during the three days of post-operative observation required coordination with other partners, with the local government borrowing 61 mattresses from the hospital, the local university, and the local refugee camps.

### Patient mobilization

Using the line listing of men with scrotal swelling identified in earlier patient estimates from the seven targeted districts, health extension workers (HEWs) informed all patients in their catchment areas (250–500 households) that they could receive hydrocele surgery free of charge at Assosa hospital during the camp. HEWs scheduled patients for confirmatory screening by trained clinical health workers at nearby health centers. This patient mobilization and screening strategy successfully located 252 out of the 385 patients with scrotal swelling (65%), with some patients not at home during the HEW visit or having moved to another area (See S1 Fig). Of the 133 patients not located, information on health center appointments was left with family members; however, none showed up for the subsequent confirmatory screening. Of the 252 patients located, 208 (82%) were diagnosed as having significant scrotal swelling consistent with hydrocele and referred to camp surgeons; for the other 44, the swelling was not due to hydrocele.

The Regional Health Bureau used radio announcements in the local language and banners to mobilize suspected hydrocele cases that were missed during the original patient estimations. An additional 70 patients with scrotal swelling who were not originally registered during the patient estimates self-referred themselves to the camp.

### Pre-operative screening

Surgeons used Capuano’s scale to classify the hydrocele by both grade and stage [13] and assessed patients for other co-morbidities such as hernia, hypertension, diabetes, severe asthma, bleeding tendency, HIV/AIDS, chronic cough, and history of previous surgery. Hospital staff also took vital signs and performed laboratory tests for hematocrit, glucosuria, urinary tract infections, blood type, and HIV. Of the 278 people assessed by surgeons, 175 (63%) met the inclusion criteria for surgery, e.g. were over 13 years of age, were diagnosed with hydrocele or hydrocele and hernia, and did not have other critical clinical conditions or serious co-morbidities. The 103 patients that were excluded from hydrocele surgery were either treated at the hospital or returned home. Reasons for exclusion included a differential diagnosis of hernia or other scrotal issues (38 patients), ineligible due to serious co-morbidity (10 patients), hydrocele surgery needing general anesthesia (2 patients), or no reason given beyond not having hydrocele (63 patients).

### Training

The camp provided an opportunity to standardize surgical and post-surgical follow-up methodologies and reinforce skill sets through supervised repetition among the surgeons and IESOs. Three urologists and a university consultant general surgeon previously trained under the USAID-funded Morbidity Management and Disability Prevention (MMDP) Project using its Filaricele Surgery Training Package [22], facilitated a five-day training for three general surgeons and five IESOs. Facilitators used the Filaricele Surgery Training Package which includes didactic lectures with instructional videos, surgical training on the Filaricele Anatomical Surgical Task Trainer (FASTT) surgical simulator, and supervised live surgery. Under close supervision of the facilitators, four teams of two trainees each operated on three to five patients a day over a period of four days for an overall total of 69 patients. Surgical procedures followed standard Ethiopia hydrocele surgery guidance; specific technical details are available in S1 Text.

Twenty clinical health workers from the communities where the patients lived also were trained for one day on the signs and symptoms of surgical site infection, how to inspect and clean surgical wounds, how to perform a sterile change of dressing, as well as how to counsel patients on adherence to post-operative follow-up.

### Post-operative care and follow-up

Following WHO guidance, patients remained for three days as in-patients after the operation under the observation of the hospital nursing staff and the operating surgeons [4]. The surgeons made daily rounds to observe patients’ conditions while the nurses performed sterile changes of Turban dressing on the third day. Nurses administered a daily antibiotic regimen of 500mg amoxicillin and 500mg metronidazole every eight hours for three days and 50 mg diclofenac every eight hours as needed to relieve pain. Patients were given antibiotics to take home at discharge to complete the seven-day course, in-line with the recommendations in the national hydrocele surgical handbook predicated on observed high rates of post-operative infections in similar setting when antibiotics were not prescribed, particularly once patients returned to their communities.

Before discharge on the third day, all patients received counseling and a wound care flashcard which included information on taking post-operative prescribed medicines, monitoring for possible post-operative complications, and guidance on when to seek medical attention. The illustrated flashcard, written in Amharic, also included an appointment card, filled out by the nurse, which referenced the exact appointment dates when each patient should present to the clinical health officers in their districts for follow up (aligned with post-operative Day 5, 7, 14, 1 month, 3 month, 6 month, and 9–12 month national recommendations, see S2 Fig flowchart).

After discharge, monitoring responsibilities for the patient devolved to the clinical health workers in the patient’s community who had attended the aforementioned one-day training. If the patient did not present to his assigned clinical health worker at the scheduled date, then the clinical health worker performed a home visit. In case of complications, such as low-grade fever or a wet wound found during a change of dressing, the clinical worker administered the same course of antibiotics given pre-and post-operatively (500mg amoxicillin and 500mg metronidazole) and changed the dressing daily. If the infection was severe, the clinical worker referred the patient back to Assosa hospital. Since no culture or sensitivity tests were available at the hospital, observations were limited to clinical signs only. The clinical workers reported their observations and actions via phone calls to the Regional Health Bureau’s Neglected Tropical Disease focal person on each of the follow-up dates.

During the planning phase of the surgery camp, the MMDP Project, the Beneshangul-Gumuz RHB and Ethiopian Surgical Society set a financially and logistically reasonable goal to actively follow-up at least 40% of the patients assessed on Day 5 at the 9–12 month benchmark. Sixty-three (41%) of the 154 patients followed up on Day 5 were randomly selected by project staff for follow up using the RANDNUM function in MS Excel. Assigned clinical health workers then interviewed selected patients using a questionnaire adapted from World Health Organization Disability Assessment Schedule 2.0 [23] and the West African Lymphatic Filariasis Morbidity Project Surgical Handbook [24]. The survey tool included questions about economic status and quality of life, asking about patients’ conditions both before surgery and after surgery.

### Costing

Costs for mobilization of patients with scrotal swelling from the patient estimate line listing, screening by community health workers, patient travel and hospital fees, and the costs of follow-up within the patients’ communities were collected from budgets from the USAID-funded MMDP Project reports. The related budget line items were summed and then divided by the total number of patients who received hydrocele surgery to get an average cost per patient. Training costs were not included as surgeon training is not necessarily a part of every hydrocele surgery camp.

### Data entry and analysis

The medical history form used by Assosa hospital was customized to collect key information on the hydrocele and peri-operative procedures, including patient’s demography, scrotal swelling, medical history including co-morbidities, surgical history, habits (alcohol, *khat* chewing, smoking), physical examination findings including stage and grading of hydrocele, pre-operative laboratory investigations, and management plan. The post-operative patient follow-up form recorded information on patient demography, characteristics of the removed hydrocele, surgical procedure, any intra-operative surgical complications, type of anesthesia administered, date and type of surgery performed, medications given, immediate complications observed (Days 0–3), duration of hospitalization, and general comments. The operating surgeons filled out both the medical history form and the patient follow-up forms and hand-delivered them to the study team at the end of each day. The 9-12-month quality of life questionnaire was paper-based and included questions concerning demographics and the social, economic and mental well-being of the patient.

The pre-coded variables were entered into EpiData3.1, cleaned, transformed and then exported to SPSS Version 21 for further analysis. The mean and standard deviation of descriptive statistics were calculated, and chi-squared tests were used to test for associations between demographic statistics and clinical findings. The Wilcoxson Signed Rank test was used to assess changes in health status, weekly income and unworked days before and after surgery.

## Results

A total of 175 patients received hydrocele surgery: 69 of 175 (39.4%) during the training and 106 of 175 (60.6%) during the camp. Average patient age was 59 years, with a range from 17 to 101 years. The presence of hydrocele averaged six years and ranged from three months to 30 years. Most patients (60.6%) presented with unilateral hydrocele (Table 1). One-hundred-and-sixty-eight patients (96%) had hydrocele alone while two (1.1%) had a recurrent hydrocele and five (2.9%) had hydrocele with hernia (Table 2). Almost 90% of operated patients had stages II or III hydrocele. Three-quarters (74.9%) of hydrocele were classified as grade 0 of burial of the penis while 17.1% were grade 1 and 2. There was a statistically significant association between age group of the patient and type of hydrocele (X^2^ = 5.428, p = 0.020); older age was associated with a higher proportion of bilateral hydrocele whereas younger age with a higher proportion of unilateral hydrocele.

**Table 1:** Characteristics of operated patients by type and stage of hydrocele.

Characteristic	Number N = 175	%
*Type of Hydrocele*		
Unilateral	106	60.6
Bilateral	69	39.4
*Diagnosis*		
Hydrocele	168	96.0
Recurrent hydrocele	2	1.1
Hydrocele with hernia	5	2.9
*Stage of Hydrocele*		
I (less than tennis ball, 160 mL)	10	5.7
II (larger than stage 1, lower pole does not extend below mid-thigh)	107	61.1
III (Lower pole extends below mid-thigh but not below upper edge of patella)	49	28.0
IV (Lower pole extends below patella and not below tibial tuberosity)	8	4.6
V (Lower pole extends below the tibial tuberosity but not below mid leg)	1	0.6
VI (Lower pole extends below mid leg)	0	0
*Grade*		
0 (No visible burial of penis, no shortening of penis)	131	74.9
1 (Partial burial of penis with visible length at least 2 cm)	17	9.7
2 (Partial burial of penis with visible length at less than 2 cm)	13	7.4
3 (Total burial of penis with glans or prepuce visible)	4	2.3
4 (Total burial of penis with stretched skin of prepuce causing problems with urination)	10	5.7

**Table 2:** Operative findings of hydrocele.

Description	Number	%
Uncomplicated hydrocele*	150	85.7
Hydrocele with pus	8	4.6
Hydrocele with hernia*	5	2.9
Hydrocele with hematoma	4	2.3
Hydrocele with hematoma and atrophied testis	2	1.1
Hydrocele with pus and hematoma	1	0.6
Hydrocele with cyst	2	1.1
Recurrent hydrocele*	2	1.1
Hydrocele with inguinal lipoma	1	0.6
**Total**	**175**	**100.0**

*Diagnosed pre-surgery and confirmed intraoperatively

Upon initiating surgery, 150 of 175 patients (85.7%) were found to have uncomplicated hydrocele. The remaining patients that presented with different operative findings are detailed in Table 2.

All patients were discharged at Day 3 after evaluation and change of dressings and there were no observed complications. In total, 21 of the 175 patients (12%) had post-operative complications, occurring between Day 3 and Day 14, with no complications observed at the one-month follow up benchmark (Table 3). At each follow-up benchmark, not all patients were followed up due to their absence from the home during the clinical health officer’s visit. It is unknown if the clinical health officers made return visits for attempted follow-up or only visited the patient one time at each benchmark. The percent of patients who attended the clinic for follow up versus the percent that were visited at home by the clinical health officers is also unknown. No patients reported to the clinical health officers at the 3-month and 6-month benchmarks.

**Table 3:** Summary of post-operative complications and management.

Time	Number (%) of Patients Followed Up	Documented Complications	Steps Taken
Day 3 (hospital discharge day)	175 (100%)		Change of dressing
Day 5	154 (88%)	1 of 154 (0.6%) patients had inability to urinate	Catheter applied by clinical health worker
Day 7	167 (95%)	1 of 167 (0.6%) patients with hematoma and 9 (5.4%) with infections	Hematoma case and 7 of the infections referred to and treated at Assosa hospital. 2 infections treated at the local health center with change of antibiotics from 500mg amoxicillin and 500mg metronidazole every 8 hours to 500 mg oral Ciprofloxacin every 12 hours for 7 days and sterile wound care.
Day 14	153 (87%)	10 of 153 (6.5%) new patients with minor infections	All treated at local health center with change of antibiotics and sterile wound care.
1 month	145 (83%)	No complications Documented	
9–12 months	63 (41%)*	1 lymph scrotum	Referred and treated at Assosa hospital

*Random sample of the 154 patients followed up on Day 5.

At the 9-12-month benchmark, the 63 patients followed were found to be recovered with no complications except for one patient with a lymph scrotum, a thickening of the scrotal skin with rupturing superficial lymphatic vesicles and draining lymph fluid, which may or may not be linked to the surgery. Five of the 63 patients randomly selected were among the 19 patients who had a post-surgical complication between the Day 5 and the 1-month follow-up benchmarks. The quality of life survey showed 100% of the patients interviewed were satisfied with their surgery. Almost all patients reported satisfaction in the ability to do work (96.8%) and increased comfort level going to their church or mosque (92.06%) and visiting friends (90.32%) (Table 4). Patients’ self-report of their health status increased from 22% stating their health status was ‘very good’ prior to surgery to 81% reporting this after surgery. The difference in health status before and after surgery was statistically significant (Z = -5.675, p = 0.000). Economic outcomes also improved overall for these patients. Before surgery, 79% of these patients reported earning less than 100 birr a week, while at 9–12 month follow up only 30% reported earning less than 100 birr a week. The difference in weekly income before and after surgery was also significant (Z = -3.175, p = 0.001). In addition, the percentage of patients who had no unworked days due to illness in the prior three months increased from 8% to 76% after surgery. There was a statistically significant difference in the number of unworked days for patients just before hydrocele surgery and 1 year after the surgery (Z = -3.346, p = 0.001).

**Table 4:** Quality of Life Survey Results at 9–12 Month Follow-up.

Quality of Life	All patients followed up (N = 63)	Patients with post-surgical infection between Day 5 and 1 month (N = 5)
**General satisfaction with the surgery**		
No problem after the operation and satisfied with the surgery	60 (95.24%)	5 (100%)
Had some problems after the operation but satisfied with the surgery	3 (4.76%)	
**Satisfaction with general health status after surgery**		
Satisfied	63 (100%)	5 (100%)
Neither satisfied nor dissatisfied		
Dissatisfied		
**Negative feelings after the surgery (n = 62)**		
Always		
Sometimes	7 (11.29%)	1 (20%)
Never	55 (88.71%)	4 (80%)
**Satisfaction with ability of working after surgery**		
Satisfied	61 (96.83%)	5 (100%)
Neither satisfied nor dissatisfied	1 (1.59%)	
Dissatisfied	1 (1.59%)	
**Ability to work after surgery**		
Better after the surgery	60 (96.77%)	5 (100%)
Same as before the surgery	2 (3.23%)	
**Economic situation after the surgery**		
Better than before the surgery	55 (90.16%)	5 (100%)
No difference	6 (9.84%)	
**Comfort level going to the church/mosque**		
Better than before the surgery	58 (92.06%)	4 (80%)
No difference	4 (6.35%)	1 (20%)
Declined to respond	1 (1.59%)	
**Comfort level to visit friends**		
Better than before the surgery	56 (90.32%)	4 (80%)
No difference	6 (9.68%)	1 (20%)
Worse than before		
**Frequency of visiting friends after surgery**		
More frequently	39 (61.90%)	5 (100%)
About the same	17 (26.98%)	
Less frequently	7 (11.11%)	
**Relationship with family and relatives after the surgery**		
Satisfied	51 (82.26%)	5 (100%)
Neither satisfied nor dissatisfied	11 (17.74%)	
**Sexual relationship after the surgery**		
Better than before the surgery	50 (81.97%)	4 (80%)
No difference	2 (3.28%)	
Worse than before	1 (1.64%)	
Declined to respond	8 (13.11%)	1(20%)

### Costing

The overall budget was US$33,652, or a cost of approximately US$192 per patient operated. Costs were highest in the hydrocele camp activity, which included the accommodation, travel and meal costs for the surgeons and surgery support staff (Table 5).

**Table 5:** Cost Breakdown by Major Activity.

Activity	Total (USD)	%
Patient mobilization	342	1.01%
Screening of patients with scrotal swelling by clinical health workers	1,056	3.13%
Hydrocele camp	22,996	68.30%
Hospital fee and travel for patients	7,488	22.50%
Follow up of patients by clinical health workers	1,770	5.25%
**Total**	**33,652**	**100.00%**

## Discussion

Hydrocele is the most common manifestation of LF morbidity, and, often the most difficult to address. Investigating novel ways to provide high-quality surgical services close to patients may help improve surgery outcomes and contribute to the achievement of LF elimination. Implementing a hydrocele surgery camp with the goal of addressing the three delays in accessing appropriate surgical care addressed this need with varying degrees of success.

To some extent, the camp approach addressed the delay in seeking care due to lack of awareness by using the lowest tiers of community health workers—HEWs and clinical health officers—to mobilize and diagnose patients. However, fewer than 50% of the patients with scrotal swelling that were referred to the camp were ultimately eligible for surgery. Further research would be necessary to compare the outcomes and costs of the community health worker approach against an approach using higher-tiered medical professionals for an initial screening. This data was not available for comparison for this article.

Conversely, of the 70 self-referred patients, 56 (80%) were approved and had surgery. Of these, 50 (72%) hailed from the three districts neighboring Assosa town. Residents of these districts spend significant amounts of time in town where a patient estimate exercise was not conducted. Thus, these 50 patients may have been missed altogether during the patient line listing. National programs should be prepared to provide surgical services to more patients than registered, particularly in urban areas that might not have required mass drug administration. Anecdotal evidence shows that hydrocele patients often do not disclose their condition during patient estimate activities until surgical services are explicitly offered. A careful cross-comparison of these 70 patients versus the patient line-listings at the community level might lend greater insight into this issue but was not included within the parameters of the intervention.

The delay in reaching care was addressed by the camp paying for patient transport to the regional hospital and for the procedure itself; however, these are not routine or sustainable interventions. On a national level, this delay will continue to exist for surgical access until planned provision of more IESO personnel or general surgeons to lower-tier hospitals is completed. However, it is important to note here the advocacy power that camps, which demonstrate to government officials the burden of hydrocele in their communities, can have to address this delay. Following this camp and subsequent presentations of its results at national forums, the neighboring and much larger region of Oromia issued a circular to all hospitals within the region to provide free hydrocele surgery services, including transport. Similarly, Tigray region added hydrocele surgery to its community health insurance scheme.

The camp addressed the third delay, the delay in receiving care, most effectively. The provision of 175 surgeries in one hospital within a two-week time frame, in a country where hospitals usually provide an average of one hydrocele surgery a month, demonstrates the success of the camp from the perspective of sheer output. Conducting a hydrocele surgical training, strengthened via initial practicum using the Filaricele Surgery Training Package, followed immediately by a surgical camp also proved a viable strategy to standardize quality care. At a national level, the SaLTS technical working group recommended including the Filaricele Surgery Training Package in the national medical school curriculum. As of February 2019, medical professors from all major medical schools attended a Filaricele Surgery Training using the FASTT simulator and six universities have rolled out the new hydrocele surgery curriculum.

No complications were reported in the three days following surgery, when patients were still in the hospital. Once released from hospital, however, post-operative complications–the majority of which were surgical site infections possibly from nosocomial infection—occurred in 12% of patients. No data on rate of complications is available for routine hydrocele surgery in Ethiopia. However, a similar hydrocele surgery camp in Nigeria which conducted 301 hydrocele surgeries, 94% of which were done using the eversion technique, reported 3.7% occurrence of immediate post-operative hematoma and 3% immediate post-operative wound infection or breakdown, with patients followed up from one to seven days post-surgery [11]. An assessment of 3,000 hydrocele resection surgeries across 10 African countries found the average occurrence of post-operative complications between 5% and 7% [5]. However, the 3,000 patients were all kept at the hospital until Day 5 which may have helped reduce post-operative infection. The primary hospital in the study area was not equipped to perform culture and sensitivity tests for severe wound infections. Hence, the surgeons managed by changing the treatment regimen by prescribing a broad-spectrum antibiotic. To address concerns about post-operative infections occurring after release from the hospital, the clinical workers training on post-hydrocele surgery was updated and now includes improved modules focusing on aseptic bandage application, with an emphasis on providing more information to patients during counseling sessions, including on the importance of post-operative hygiene. Further research in Ethiopia and elsewhere would be useful to determine how the rate of post-operative complications after a surgical camp compares with the rate of complications after hydrocele surgeries in a standard hospital setting. Further research on how to reduce the rate of post-operative infections after hospital discharge would also be useful in improving surgical outcomes.

Clinical health officers based in the community health centers successfully followed up with at least 89% of patients on the Day 5, 7 and 14 benchmarks. Part of this success is because clinical health officers were paid per diem to ensure that sufficient data were collected. Given that reports fell off completely after the one-month benchmark, this follow-up may not be feasible without some sort of financial incentive. Since this pilot was completed, hydrocele surgery activities have expanded into other regions where per diem was not offered for follow-up. In these new areas, information on the benchmarks after patients are released from the hospital has been very slow in arriving to the regional and national levels. These anecdotal findings may suggest that the follow-up guidelines are too difficult to integrate in the established health system effectively without additional support, such as per diem allocation for patient follow-up and reporting or building surgical patient follow-up into the general roles and responsibilities of the clinical health worker or incentivizing clinical health workers to visit patients at home.

The quality of life data collected at the 9–12 month benchmark is a further indicator that the patients deemed the surgeries successful; 95% of the surveyed patients reported satisfaction with the surgery with no problems. Furthermore, 100% of the surveyed patients reported an improvement in their general health status after the surgery, with economic indicators and ability to work all increasing for almost all patients. Similarly, for the five patients interviewed that had a post-surgical infection during the Day 5 and 1-month follow-up, all five reported satisfaction with the surgery and general improvement in general health status.

The US$193 cost per patient is significant for what is normally an out-patient procedure in Ethiopia. However, it fits well below global standards for cost-effectiveness of hydrocele surgery, which is estimated as high as US$360-US$398 [25,26]. There are limitations to this cost estimate; however, as no data was collected from the perspective of the patients or the host government. While patient travel was covered by donor funds, other costs to patients could have included travel for the patients’ families and regular meals for the patients themselves. In-kind investments, such as radio messages, two vehicles for patient transport, extra mattresses, subsidized meals, operating theaters, and time of staff for coordinating, preparing and implementing the camps, from the FMOH and Regional Health Bureau also were not quantified.

The surgical camp required a large investment of time, staff, and external and in-kind funding that is likely not sustainable (or necessary once the majority of hydrocele patients are addressed in a given area) over the long-term. For example, the several advocacy meetings led by the Surgical Society of Ethiopia and the FMOH, with the Regional Health Bureau, Assosa Zonal Health Department, and Assosa hospital were critical to ensure allocation of nursing, anaesthesiology, and cleaning staff, as well as sufficient medical supplies. The recruitment of surgeons from hospitals around the country was challenging, as many hospitals could not release their only resident surgeon for more than a week. For this reason, a large training and camp approach may be difficult to replicate frequently in countries with similar human resource challenges.

However, the camp was effective at providing a large number of quality surgeries in a short time. The use of peripheral health workers to mobilize and follow up patients helped address delays in seeking and receiving quality care. In addition, the camp approach brought hydrocele surgery needs to the attention of communities, hospital management, and national authorities, resulting in changes which made hydrocele surgery more affordable within Ethiopia.

## Supporting information

S1 Fig Surgical Procedures. (TIF)

S2 Fig Ethiopia hydrocele surgery post-operative flowchart. (TIFF)

S1 Text Surgical Procedures. (DOCX)
