# Supplementary figures and images for: The implementation and impact of a pilot hydrocele surgery camp for LF-endemic communities in Ethiopia
Source: PLoS Negl Trop Dis. 2021 Oct 25;15(10):e0009403. doi: 10.1371/journal.pntd.0009403 (PMC8568282; doi:10.1371/journal.pntd.0009403)

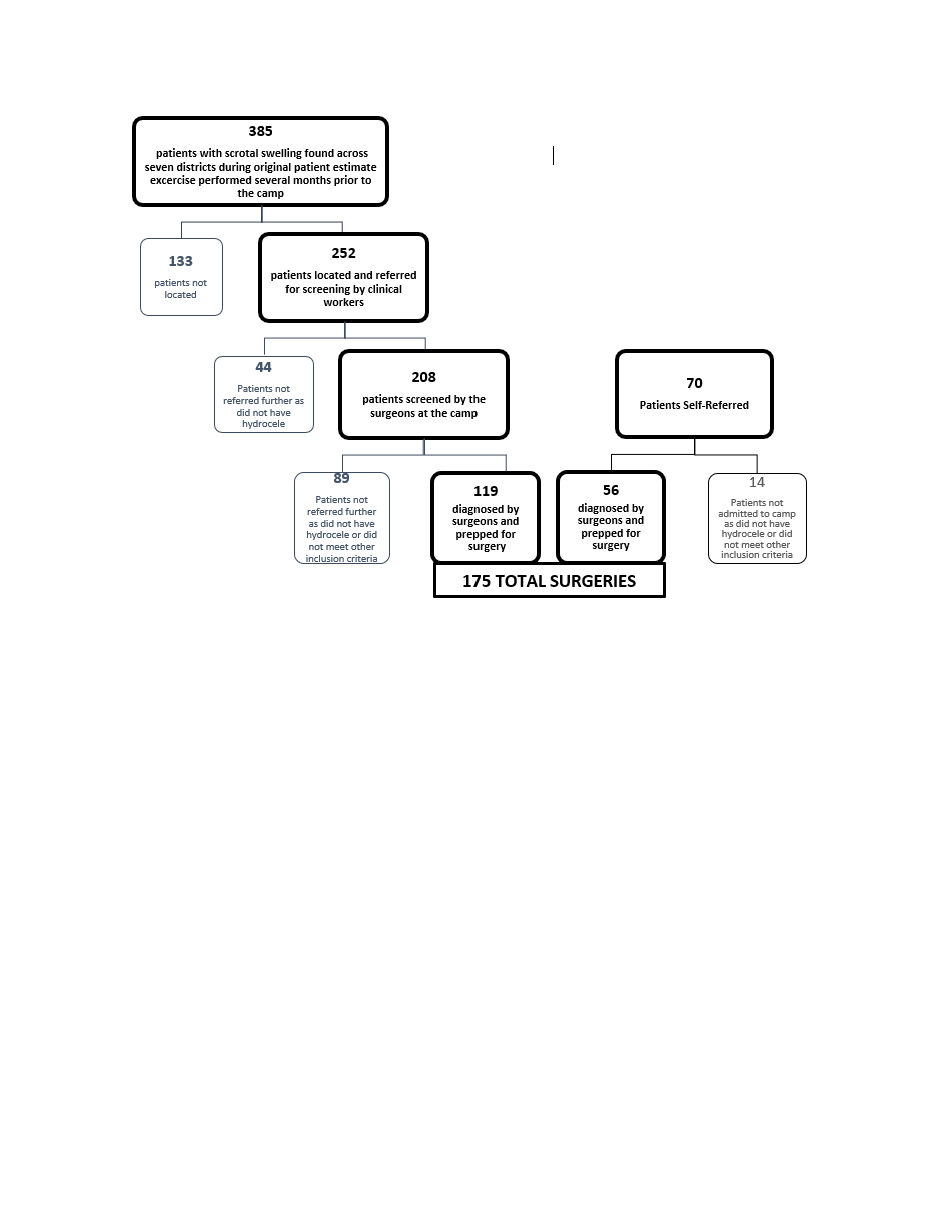

Supplement: S1 Fig — (TIF) [file pntd.0009403.s001.tif]

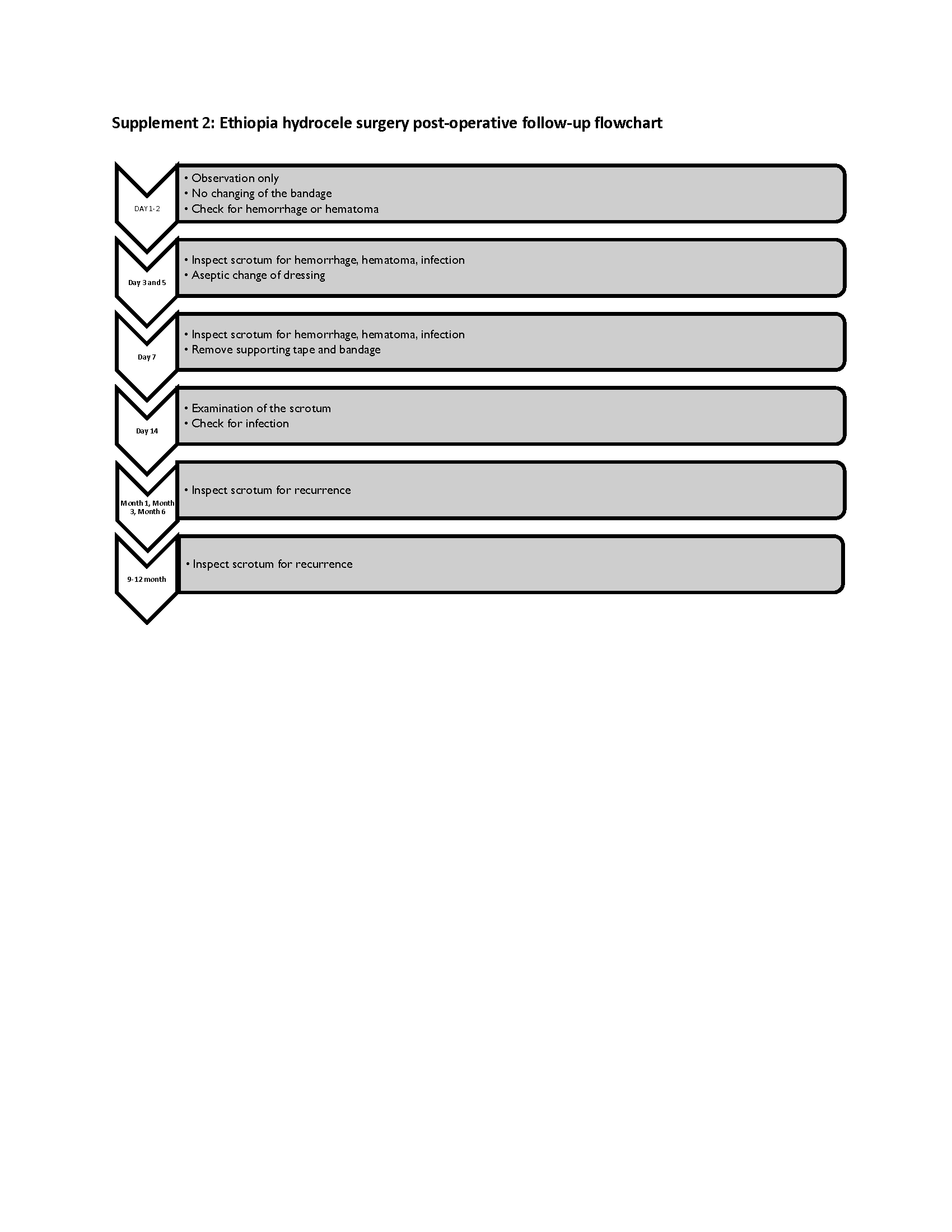

Supplement: S2 Fig — (TIFF) [file pntd.0009403.s002.tiff]
