# Supplementary material for: The implementation and impact of a pilot hydrocele surgery camp for LF-endemic communities in Ethiopia
Source: PLoS Negl Trop Dis. 2021 Oct 25;15(10):e0009403. doi: 10.1371/journal.pntd.0009403 (PMC8568282; doi:10.1371/journal.pntd.0009403)
Supplement: S1 Text — (DOCX) [file pntd.0009403.s003.docx]

**S1 Text. Surgical Procedures**

*Surgical Screening*

During the initial surgical screening, the surgeons checked for any scrotal ulceration or surgical scars and evaluated the diameter, stage and grade of the scrotal swelling, using Capuano’s proposed classification [1,2]. Hydroceles were classified into six stages according to their size with Stage I compared to less than the size of a tennis ball and Stage VI occurring when the lower pole of the scrotum reached between the middle of the leg and the ankle. The burial of the penis was classified from Grade 0 to Grade 4 with Grade 0 representing no burial of the penis and Grade 4 representing total burial with the glans penis not visible [1].

*Surgical Preparation*

Patients considered fit for surgery were counseled on the next day’s procedure and gave written informed consent in Amharic as per hospital regulations. Relatives or health workers translated for patients who only spoke local dialects. Patients took amoxicillin and metronidazole orally the night before surgery and showered before going to sleep. Patients were not allowed to eat or drink after a light meal the evening before surgery.

On the morning of the surgery, patients were given 500mg amoxicillin and 500mg metronidazole. Once in the operating theatre, patients received an intravenous saline solution. Before skin preparation, the surgeons thoroughly washed the scrotum and genital area with soap and water. They also carefully cut the pubic hair using scissors. Sterile surgical drapes were placed after the surgical site was cleaned with povidone iodine.

*Operative Procedures*

As determined by the surgeons, either local (lidocaine with adrenaline and sedation by diazepam) anesthesia was administered by the lead surgeon, or spinal (bupivacaine 0.5%) anesthesia was administered by the anesthetists. The anesthetist monitored the patient’s condition throughout the procedure to ensure each patient was free of pain and did not fall into hypovolemia or hypoglycemia during the procedure.

Surgeons used a scrotal median raphe incision for cutting the skin, Dartos muscle, and other layers. After blunt dissection and extraction of the hydrocele sac (tunica vaginalis), surgeons removed the hydrocele fluid and excised the tunica vaginalis leaving a one-centimeter border around the testis. Hemostasis was secured by applying an interlocking stitch around the cut edge of the tunica vaginalis. The surgeons then closed the wound in layers using 3/0 vicryl suture ensuring no remaining scrotal drainage.

References:

1. Capuano GP, Capuano C. Surgical management of morbidity due to lymphatic filariasis: the usefulness of a standardized international clinical classification of hydroceles. Trop Biomed

[Internet]. 2012 Mar;29(1):24–38. Available from:

<http://www.ncbi.nlm.nih.gov/pubmed/22543600>

2. World Health Organization. Report of an informal consultation: Surgical Approaches to the urogenital manifestations of lymphatic filariasis. Geneva, Switzerland; 2002.
